# (Di-2-pyridyl­amine-κ^2^ *N*,*N*′)[hydro­tris­(3,5-diphenyl­pyrazol-1-yl-κ*N* ^2^)borato]nickel(II) bromide dichloro­methane monosolvate

**DOI:** 10.1107/S1600536811010233

**Published:** 2011-03-23

**Authors:** David J. Harding, Phimphaka Harding, Harry Adams

**Affiliations:** a Molecular Technology Research Unit, School of Science, Walailak University, Thasala, Nakhon Si Thammarat 80161, Thailand; b Department of Chemistry, Faculty of Science, University of Sheffield, Brook Hill, Sheffield S3 7HF, England

## Abstract

In the title compound, [Ni(C_45_H_34_BN_6_)(C_10_H_9_N_3_)]Br·CH_2_Cl_2_, the Ni^II^ atom is five-coordinated by the tridentate hydro­tris­(3,5-diphenyl­pyrazol­yl)borate ligand and a bidentate di-2-pyridyl­amine ligand in a distorted square-pyramidal geometry. In the crystal, inter­molecular N—H⋯Br and C—H⋯Br hydrogen bonds link the Ni complex cations and the Br^−^ ions, forming a chain along the *c* axis.

## Related literature

For related structures of Ni^II^ complexes with substituted tris­(pyrazol­yl)borate ligands, see: Harding *et al.* (2007 ▶, 2010 ▶); Uehara *et al.* (2002 ▶); Trofimenko (1999 ▶). For related structures of Ni^II^ complexes with dipyridyl­amine, see: Lu *et al.* (2001 ▶); Rahaman *et al.* (2005 ▶); Uddin *et al.* (1997 ▶). For a discussion on trigonality in five-coordinate complexes, see: Addison *et al.* (1984 ▶).
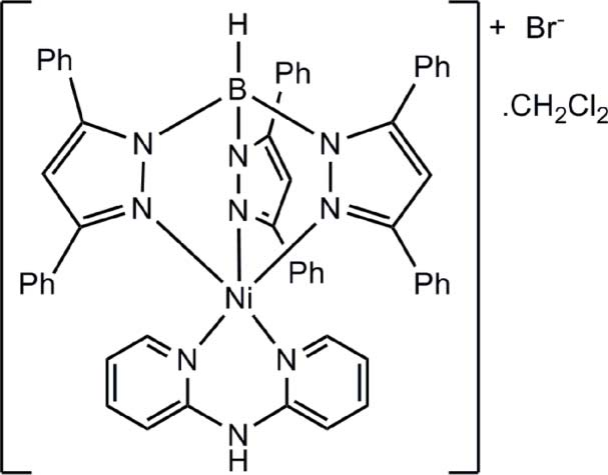


## Experimental

### 

#### Crystal data

[Ni(C_45_H_34_BN_6_)(C_10_H_9_N_3_)]Br·CH_2_Cl_2_
*M* *_r_* = 1064.34
Triclinic, 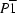

*a* = 10.3263 (13) Å
*b* = 13.9040 (16) Å
*c* = 17.622 (2) Å
α = 91.314 (6)°
β = 99.245 (6)°
γ = 101.968 (6)°
*V* = 2438.8 (5) Å^3^
*Z* = 2
Mo *K*α radiation
μ = 1.38 mm^−1^
*T* = 150 K
0.39 × 0.38 × 0.32 mm

#### Data collection

Bruker APEXII CCD diffractometer
Absorption correction: multi-scan (*SADABS*; Bruker, 1997 ▶) *T*_min_ = 0.616, *T*_max_ = 0.667
44352 measured reflections
11127 independent reflections
9003 reflections with *I* > 2σ(*I*)
*R*_int_ = 0.061

#### Refinement

*R*[*F* ^2^ > 2σ(*F* ^2^)] = 0.055
*wR*(*F* ^2^) = 0.161
*S* = 1.11
11127 reflections
631 parameters
H-atom parameters constrained
Δρ_max_ = 1.40 e Å^−3^
Δρ_min_ = −1.66 e Å^−3^

### 

Data collection: *APEX2* (Bruker, 2008 ▶); cell refinement: *SAINT* (Bruker, 2008 ▶); data reduction: *SAINT*; program(s) used to solve structure: *SHELXS97* (Sheldrick, 2008 ▶); program(s) used to refine structure: *SHELXL97* (Sheldrick, 2008 ▶); molecular graphics: *X-SEED* (Barbour, 2001 ▶); software used to prepare material for publication: *publCIF* (Westrip, 2010 ▶).

## Supplementary Material

Crystal structure: contains datablocks I, global. DOI: 10.1107/S1600536811010233/is2690sup1.cif

Structure factors: contains datablocks I. DOI: 10.1107/S1600536811010233/is2690Isup2.hkl

Additional supplementary materials:  crystallographic information; 3D view; checkCIF report

## Figures and Tables

**Table 1:** Selected bond lengths (Å)

Ni1—N2	2.006 (2)
Ni1—N8	2.024 (2)
Ni1—N7	2.032 (2)
Ni1—N6	2.072 (2)
Ni1—N4	2.078 (2)

**Table 2:** Hydrogen-bond geometry (Å, °)

*D*—H⋯*A*	*D*—H	H⋯*A*	*D*⋯*A*	*D*—H⋯*A*
N9—H9⋯Br1^i^	0.88	2.41	3.265 (2)	165
C52—H52⋯Br1^i^	0.95	2.92	3.706 (3)	140
C31—H31⋯Br1^ii^	0.95	2.91	3.858 (3)	172

Symmetry codes: (i) 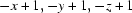; (ii) 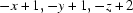.

**Fig. 1.**
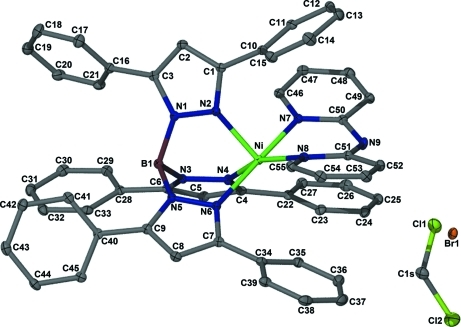
The molecular structure of the title compound, showing the atom-labelling scheme. Hydrogen atoms have been removed for clarity. Displacement ellipsoids are drawn at the 30% probability level.

**Fig. 2.**
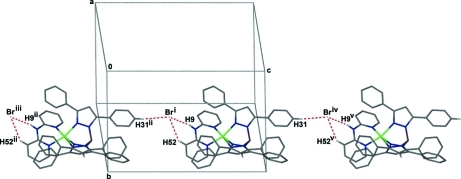
The molecular packing in the title compound, showing the N—H···Br and C—H···Br interactions that form the one-dimensional chains in the structure. Only selected atoms are labelled or shown for clarity [Symmetry codes: (i) 1 - x, 1 - y, 1 - z; (ii) x, y, z - 1; (iii) 1 - x, 1 - y, -z; (iv) 1 - x, 1 - y, 2 - z; (v) x, y, z + 1].

## supplementary crystallographic information

### Comment

Tris(pyrazolyl)borates are versatile and popular ligands in coordination chemistry with many complexes now known (Trofimenko, 1999). While neutral half-sandwich complexes are common, [Tp^R^NiX] (Tp^R^ = substituted tris(pyrazolyl)borate, *X* = monoanionic ligand) cationic species are limited to those reported by Akita and co-workers and our research group (Uehara *et al.*, 2002; Harding *et al.*, 2010).

The title compound, [Tp^Ph2^Ni(dpa)]Br 1 (dpa = di-2-pyridylamine), crystallizes in the triclinic, *P*1 space group with one molecule of dicholormethane. The structure is shown in Figure 1 while important bond lengths and angles are given in the supporting tables. The nickel metal centre is five coordinate with the Tp^Ph2^ ligand κ^3^-coordinated resulting in a geometry best described as square pyramidal (τ = 0.23; Addison *et al.*, 1984). The Ni—N bond lengths for the Tp^Ph2^ ligand are typical of [Tp^R^Ni(N—N)]^+^ (N—N = neutral nitrogen donor) complexes although the difference between the apical and equatorial Ni—N bond distances is larger; ΔNi-N = 0.07 Å for 1*cf*. ΔNi-N = 0.04 Å for [Tp^Ph,Me^Ni(bpym)]PF_6_ (Harding *et al.*, 2010). The Ni—N bond lengths are also somewhat shorter than previously reported where values are between 2.029–2.121 Å (Lu *et al.*, 2001; Rahaman *et al.*, 2005; Uddin *et al.*, 1997). The dihedral angle between the two pyridine rings is 22.5° and thus the ligand adopts a boat conformation as found in other Ni-dpa compounds {21.9 and 28.1° in [Ni(dpa)_2_(N_3_)_2_] (Rahaman *et al.*, 2005); 24.7° in [Ni(dpa)_2_(acac)]NO_3_ (Uddin *et al.*, 1997)}.

The packing in the structure principally involves an N—H···Br hydrogen bond and two C—H···Br interactions (Table 2). These combine to form one-dimensional chains of nickel cations separated by the bromide anions (Fig. 2). Further chains are found above and below the chain shown with the direction of the chain alternating throughout the structure. Interestingly, the supramolecular motif exhibited by the dpa ligand towards the bromide anion in 1 has previously been reported in the structure of [Ni(oxalate)(dpa)] although in this instance the interactions are between the N—H and C—H groups of the dpa ligand and a coordinated oxygen atom of the oxalate ligand (Lu *et al.*, 2001).

### Experimental

TpNiBr (162 mg, 0.20 mmol; Harding *et al.*, 2007) was dissolved in CH_2_Cl_2_ (10 ml) giving a deep pink solution and then stirred for 5 min. di-2-pyridylamine (35 mg, 0.20 mmol) was added giving a green solution. The solution was stirred for 2 hrs and filtered through celite. The resulting green solution was layered with hexanes (30 ml). After two days red-brown crystals appeared. These were washed with EtOH (3 × 3 ml) and hexanes (2 × 5 ml) (157 mg, 74%). ν_max_(KBr)/cm^-1^ 3379, (νNH), 3066w, 3045w, (νCH), 2634w (νBH). m/*z* (ESI) 898 [M—Br^-^]^+^. Anal. Calc. for C_56_H_45_N_9_BBrCl_2_Ni [Tp^Ph2^Ni(pyNHpy)]Br.CH_2_Cl_2_: C 63.16, H 4.26, N 11.85%; found: C 63.44, H 4.08, N, 12.02%.

### Refinement

Hydrogen atoms were placed geometrically and refined with a riding model and with *U*_iso_ constrained to be 1.2 (CH, NH or BH) or 1.5 (CH_2_) times *U*_eq_ of the carrier atom. The highest residual peak and the deepest hole in the difference Fourier map are located 0.87 and 0.81 Å, respectively, from Br1.

### Crystal data

**Table:**

[Ni(C_45_H_34_BN_6_)(C_10_H_9_N_3_)]Br·CH_2_Cl_2_	*Z* = 2
*M**_r_* = 1064.34	*F*(000) = 1092
Triclinic, *P*1	*D*_x_ = 1.449 Mg m^−^^3^
Hall symbol: -P 1	Mo *K*α radiation, λ = 0.71073 Å
*a* = 10.3263 (13) Å	Cell parameters from 9896 reflections
*b* = 13.9040 (16) Å	θ = 2.2–27.6°
*c* = 17.622 (2) Å	µ = 1.38 mm^−^^1^
α = 91.314 (6)°	*T* = 150 K
β = 99.245 (6)°	Block, red
γ = 101.968 (6)°	0.39 × 0.38 × 0.32 mm
*V* = 2438.8 (5) Å^3^	

### Data collection

**Table:**

Bruker APEXII CCD diffractometer	11127 independent reflections
Radiation source: fine-focus sealed tube	9003 reflections with *I* > 2σ(*I*)
graphite	*R*_int_ = 0.061
φ and ω scans	θ_max_ = 27.8°, θ_min_ = 1.5°
Absorption correction: multi-scan (*SADABS*; Bruker, 1997)	*h* = −13→13
*T*_min_ = 0.616, *T*_max_ = 0.667	*k* = −18→18
44352 measured reflections	*l* = −23→22

### Refinement

**Table:**

Refinement on *F*^2^	Primary atom site location: structure-invariant direct methods
Least-squares matrix: full	Secondary atom site location: difference Fourier map
*R*[*F*^2^ > 2σ(*F*^2^)] = 0.055	Hydrogen site location: inferred from neighbouring sites
*wR*(*F*^2^) = 0.161	H-atom parameters constrained
*S* = 1.11	*w* = 1/[σ^2^(*F*_o_^2^) + (0.1045*P*)^2^] where *P* = (*F*_o_^2^ + 2*F*_c_^2^)/3
11127 reflections	(Δ/σ)_max_ = 0.002
631 parameters	Δρ_max_ = 1.40 e Å^−^^3^
0 restraints	Δρ_min_ = −1.66 e Å^−^^3^

### Special details

**Table:**

Geometry. All e.s.d.'s (except the e.s.d. in the dihedral angle between two l.s. planes) are estimated using the full covariance matrix. The cell e.s.d.'s are taken into account individually in the estimation of e.s.d.'s in distances, angles and torsion angles; correlations between e.s.d.'s in cell parameters are only used when they are defined by crystal symmetry. An approximate (isotropic) treatment of cell e.s.d.'s is used for estimating e.s.d.'s involving l.s. planes.
Refinement. Refinement of *F*^2^ against ALL reflections. The weighted *R*-factor *wR* and goodness of fit *S* are based on *F*^2^, conventional *R*-factors *R* are based on *F*, with *F* set to zero for negative *F*^2^. The threshold expression of *F*^2^ > σ(*F*^2^) is used only for calculating *R*-factors(gt) *etc*. and is not relevant to the choice of reflections for refinement. *R*-factors based on *F*^2^ are statistically about twice as large as those based on *F*, and *R*- factors based on ALL data will be even larger.

### Fractional atomic coordinates and isotropic or equivalent isotropic displacement parameters (Å^2^)

**Table:**

	*x*	*y*	*z*	*U*_iso_*/*U*_eq_	
Ni1	0.17841 (3)	0.77166 (2)	0.740382 (18)	0.01490 (11)	
Br1	0.71112 (3)	0.348348 (18)	0.589511 (15)	0.02187 (10)	
B1	0.1614 (3)	0.7995 (2)	0.91011 (17)	0.0167 (6)	
H1	0.1524	0.8086	0.9653	0.020*	
N1	0.0212 (2)	0.75539 (15)	0.86037 (12)	0.0154 (5)	
N2	0.0066 (2)	0.76151 (15)	0.78204 (12)	0.0163 (5)	
N3	0.2582 (2)	0.72881 (15)	0.90199 (12)	0.0154 (5)	
N4	0.2806 (2)	0.70719 (15)	0.82967 (12)	0.0175 (5)	
N5	0.2252 (2)	0.89938 (15)	0.87984 (12)	0.0164 (5)	
N6	0.2588 (2)	0.90142 (16)	0.80706 (12)	0.0169 (5)	
N7	0.1159 (2)	0.63742 (15)	0.68289 (12)	0.0159 (5)	
N8	0.1395 (2)	0.83385 (15)	0.63884 (12)	0.0172 (5)	
N9	0.1782 (2)	0.69877 (16)	0.56775 (13)	0.0201 (5)	
H9	0.2220	0.6853	0.5313	0.024*	
C1	−0.1233 (3)	0.72381 (18)	0.75315 (15)	0.0167 (5)	
C2	−0.1929 (3)	0.69319 (19)	0.81296 (15)	0.0191 (6)	
H2	−0.2858	0.6643	0.8086	0.023*	
C3	−0.0993 (3)	0.71340 (18)	0.87993 (15)	0.0172 (5)	
C4	0.3598 (3)	0.64079 (19)	0.83670 (16)	0.0181 (6)	
C5	0.3875 (3)	0.61909 (19)	0.91359 (16)	0.0193 (6)	
H5	0.4402	0.5744	0.9343	0.023*	
C6	0.3223 (3)	0.67601 (18)	0.95385 (15)	0.0172 (5)	
C7	0.3332 (3)	0.99165 (19)	0.80075 (15)	0.0183 (6)	
C8	0.3454 (3)	1.04946 (19)	0.86828 (15)	0.0204 (6)	
H8	0.3904	1.1165	0.8783	0.024*	
C9	0.2790 (3)	0.98969 (18)	0.91788 (15)	0.0182 (5)	
C10	−0.1756 (3)	0.71772 (18)	0.66903 (15)	0.0172 (5)	
C11	−0.2090 (3)	0.62684 (19)	0.62742 (15)	0.0190 (6)	
H11	−0.1958	0.5690	0.6525	0.023*	
C12	−0.2617 (3)	0.6208 (2)	0.54935 (16)	0.0230 (6)	
H12	−0.2840	0.5589	0.5212	0.028*	
C13	−0.2819 (3)	0.7046 (2)	0.51233 (16)	0.0268 (7)	
H13	−0.3166	0.7001	0.4588	0.032*	
C14	−0.2515 (3)	0.7950 (2)	0.55343 (18)	0.0300 (7)	
H14	−0.2671	0.8523	0.5284	0.036*	
C15	−0.1980 (3)	0.8013 (2)	0.63165 (16)	0.0244 (6)	
H15	−0.1767	0.8633	0.6597	0.029*	
C16	−0.1198 (3)	0.69039 (19)	0.95942 (15)	0.0194 (6)	
C17	−0.2312 (3)	0.7111 (2)	0.98612 (17)	0.0271 (7)	
H17	−0.2902	0.7434	0.9544	0.033*	
C18	−0.2570 (3)	0.6849 (2)	1.05885 (19)	0.0337 (7)	
H18	−0.3335	0.6993	1.0765	0.040*	
C19	−0.1708 (3)	0.6378 (2)	1.10556 (17)	0.0305 (7)	
H19	−0.1871	0.6210	1.1556	0.037*	
C20	−0.0605 (3)	0.6154 (2)	1.07857 (17)	0.0291 (7)	
H20	−0.0023	0.5820	1.1099	0.035*	
C21	−0.0354 (3)	0.6417 (2)	1.00588 (16)	0.0238 (6)	
H21	0.0402	0.6263	0.9878	0.029*	
C22	0.4052 (3)	0.6003 (2)	0.76987 (15)	0.0182 (6)	
C23	0.4300 (3)	0.6555 (2)	0.70647 (16)	0.0211 (6)	
H23	0.4216	0.7222	0.7069	0.025*	
C24	0.4666 (3)	0.6145 (2)	0.64297 (17)	0.0256 (6)	
H24	0.4823	0.6528	0.6001	0.031*	
C25	0.4805 (3)	0.5171 (2)	0.64191 (17)	0.0263 (7)	
H25	0.5043	0.4884	0.5982	0.032*	
C26	0.4593 (3)	0.4626 (2)	0.70501 (18)	0.0268 (7)	
H26	0.4705	0.3964	0.7047	0.032*	
C27	0.4221 (3)	0.5027 (2)	0.76876 (16)	0.0223 (6)	
H27	0.4081	0.4641	0.8117	0.027*	
C28	0.3184 (3)	0.6792 (2)	1.03712 (15)	0.0181 (6)	
C29	0.2987 (3)	0.5906 (2)	1.07436 (16)	0.0226 (6)	
H29	0.2902	0.5301	1.0459	0.027*	
C30	0.2915 (3)	0.5901 (2)	1.15211 (17)	0.0272 (7)	
H30	0.2781	0.5296	1.1766	0.033*	
C31	0.3041 (3)	0.6788 (2)	1.19455 (17)	0.0277 (7)	
H31	0.2987	0.6786	1.2479	0.033*	
C32	0.3245 (3)	0.7671 (2)	1.15847 (16)	0.0258 (7)	
H32	0.3333	0.8274	1.1873	0.031*	
C33	0.3322 (3)	0.7679 (2)	1.08051 (16)	0.0206 (6)	
H33	0.3469	0.8288	1.0564	0.025*	
C34	0.3926 (3)	1.01965 (19)	0.73153 (16)	0.0195 (6)	
C35	0.4073 (3)	1.1167 (2)	0.70869 (16)	0.0236 (6)	
H35	0.3789	1.1643	0.7379	0.028*	
C36	0.4633 (3)	1.1438 (2)	0.64353 (17)	0.0286 (7)	
H36	0.4718	1.2095	0.6280	0.034*	
C37	0.5063 (3)	1.0755 (2)	0.60158 (18)	0.0320 (7)	
H37	0.5444	1.0942	0.5571	0.038*	
C38	0.4943 (3)	0.9794 (2)	0.62384 (17)	0.0300 (7)	
H38	0.5232	0.9323	0.5944	0.036*	
C39	0.4395 (3)	0.9522 (2)	0.68946 (16)	0.0217 (6)	
H39	0.4342	0.8870	0.7056	0.026*	
C40	0.2741 (3)	1.01458 (18)	0.99895 (15)	0.0172 (5)	
C41	0.1596 (3)	0.9867 (2)	1.03282 (16)	0.0215 (6)	
H41	0.0803	0.9470	1.0038	0.026*	
C42	0.1612 (3)	1.0169 (2)	1.10893 (17)	0.0248 (6)	
H42	0.0830	0.9980	1.1316	0.030*	
C43	0.2772 (3)	1.0748 (2)	1.15167 (16)	0.0260 (7)	
H43	0.2781	1.0951	1.2036	0.031*	
C44	0.3913 (3)	1.1028 (2)	1.11900 (16)	0.0230 (6)	
H44	0.4705	1.1423	1.1484	0.028*	
C45	0.3897 (3)	1.07291 (19)	1.04271 (16)	0.0205 (6)	
H45	0.4681	1.0924	1.0202	0.025*	
C46	0.0691 (3)	0.55904 (19)	0.72256 (16)	0.0186 (6)	
H46	0.0511	0.5711	0.7727	0.022*	
C47	0.0465 (3)	0.4634 (2)	0.69425 (17)	0.0224 (6)	
H47	0.0138	0.4103	0.7239	0.027*	
C48	0.0729 (3)	0.4465 (2)	0.62035 (16)	0.0240 (6)	
H48	0.0591	0.3811	0.5990	0.029*	
C49	0.1187 (3)	0.52491 (19)	0.57875 (16)	0.0206 (6)	
H49	0.1385	0.5144	0.5288	0.025*	
C50	0.1360 (3)	0.62104 (19)	0.61122 (15)	0.0173 (5)	
C51	0.1619 (3)	0.79498 (18)	0.57285 (15)	0.0182 (6)	
C52	0.1712 (3)	0.84904 (19)	0.50696 (16)	0.0214 (6)	
H52	0.1905	0.8206	0.4616	0.026*	
C53	0.1520 (3)	0.9442 (2)	0.50915 (16)	0.0238 (6)	
H53	0.1585	0.9824	0.4654	0.029*	
C54	0.1228 (3)	0.98380 (19)	0.57604 (16)	0.0232 (6)	
H54	0.1076	1.0488	0.5785	0.028*	
C55	0.1166 (3)	0.92684 (19)	0.63817 (16)	0.0207 (6)	
H55	0.0950	0.9537	0.6834	0.025*	
C1S	0.9342 (3)	0.1996 (2)	0.72085 (19)	0.0298 (7)	
H1S1	0.9397	0.2196	0.7757	0.036*	
H1S2	0.8902	0.2455	0.6892	0.036*	
Cl1	0.83690 (9)	0.07892 (6)	0.70135 (5)	0.0398 (2)	
Cl2	1.09888 (9)	0.20725 (6)	0.70032 (6)	0.0431 (2)	

### Atomic displacement parameters (Å^2^)

**Table:**

	*U*^11^	*U*^22^	*U*^33^	*U*^12^	*U*^13^	*U*^23^
Ni1	0.0163 (2)	0.01283 (17)	0.01686 (19)	0.00606 (13)	0.00296 (14)	0.00015 (13)
Br1	0.02777 (19)	0.01687 (15)	0.02382 (17)	0.00711 (12)	0.00983 (12)	0.00051 (11)
B1	0.0167 (15)	0.0161 (14)	0.0186 (14)	0.0077 (11)	0.0019 (12)	0.0002 (11)
N1	0.0170 (12)	0.0135 (10)	0.0184 (11)	0.0083 (8)	0.0040 (9)	0.0003 (8)
N2	0.0179 (12)	0.0141 (10)	0.0180 (11)	0.0056 (8)	0.0037 (9)	−0.0014 (8)
N3	0.0151 (11)	0.0163 (10)	0.0154 (10)	0.0060 (8)	0.0008 (8)	0.0002 (8)
N4	0.0207 (12)	0.0144 (10)	0.0193 (11)	0.0071 (9)	0.0051 (9)	−0.0005 (8)
N5	0.0186 (12)	0.0165 (11)	0.0162 (10)	0.0081 (9)	0.0034 (9)	0.0000 (8)
N6	0.0189 (12)	0.0161 (10)	0.0169 (11)	0.0056 (9)	0.0040 (9)	0.0016 (8)
N7	0.0153 (11)	0.0151 (10)	0.0186 (11)	0.0068 (8)	0.0024 (9)	−0.0009 (8)
N8	0.0160 (12)	0.0152 (10)	0.0211 (11)	0.0055 (8)	0.0028 (9)	−0.0004 (9)
N9	0.0261 (13)	0.0185 (11)	0.0213 (11)	0.0123 (9)	0.0105 (10)	0.0011 (9)
C1	0.0167 (14)	0.0121 (12)	0.0226 (13)	0.0068 (10)	0.0030 (11)	−0.0012 (10)
C2	0.0165 (14)	0.0165 (12)	0.0251 (14)	0.0052 (10)	0.0041 (11)	0.0001 (10)
C3	0.0180 (14)	0.0120 (11)	0.0245 (13)	0.0089 (10)	0.0048 (11)	0.0007 (10)
C4	0.0163 (14)	0.0138 (12)	0.0253 (14)	0.0050 (10)	0.0044 (11)	−0.0002 (10)
C5	0.0178 (14)	0.0165 (12)	0.0265 (14)	0.0108 (10)	0.0027 (11)	0.0015 (10)
C6	0.0159 (14)	0.0145 (12)	0.0211 (13)	0.0053 (10)	0.0004 (11)	0.0011 (10)
C7	0.0189 (14)	0.0144 (12)	0.0234 (14)	0.0087 (10)	0.0023 (11)	0.0027 (10)
C8	0.0215 (15)	0.0153 (12)	0.0243 (14)	0.0062 (10)	0.0008 (11)	0.0006 (10)
C9	0.0200 (14)	0.0129 (12)	0.0233 (13)	0.0083 (10)	0.0024 (11)	−0.0006 (10)
C10	0.0151 (14)	0.0165 (12)	0.0211 (13)	0.0061 (10)	0.0033 (11)	−0.0002 (10)
C11	0.0156 (14)	0.0178 (13)	0.0247 (14)	0.0080 (10)	0.0013 (11)	−0.0010 (11)
C12	0.0223 (15)	0.0237 (14)	0.0245 (14)	0.0098 (11)	0.0037 (12)	−0.0063 (11)
C13	0.0294 (17)	0.0305 (16)	0.0190 (14)	0.0055 (13)	0.0007 (12)	−0.0002 (12)
C14	0.0379 (19)	0.0219 (14)	0.0289 (16)	0.0089 (13)	−0.0022 (14)	0.0074 (12)
C15	0.0279 (16)	0.0156 (13)	0.0277 (15)	0.0041 (11)	0.0002 (12)	−0.0006 (11)
C16	0.0226 (15)	0.0144 (12)	0.0220 (13)	0.0050 (10)	0.0047 (11)	0.0003 (10)
C17	0.0296 (17)	0.0285 (15)	0.0284 (15)	0.0145 (13)	0.0089 (13)	0.0059 (12)
C18	0.0361 (19)	0.0382 (18)	0.0349 (17)	0.0157 (15)	0.0188 (15)	0.0074 (14)
C19	0.0322 (18)	0.0357 (17)	0.0228 (15)	0.0042 (14)	0.0057 (13)	0.0068 (13)
C20	0.0241 (16)	0.0337 (17)	0.0276 (16)	0.0031 (13)	0.0010 (12)	0.0115 (13)
C21	0.0187 (15)	0.0257 (14)	0.0273 (15)	0.0064 (11)	0.0023 (12)	0.0042 (12)
C22	0.0124 (13)	0.0213 (13)	0.0227 (13)	0.0100 (10)	0.0003 (10)	−0.0020 (10)
C23	0.0172 (14)	0.0217 (13)	0.0273 (14)	0.0098 (11)	0.0053 (11)	0.0013 (11)
C24	0.0224 (16)	0.0322 (16)	0.0254 (15)	0.0104 (12)	0.0070 (12)	0.0021 (12)
C25	0.0197 (15)	0.0327 (16)	0.0282 (15)	0.0106 (12)	0.0037 (12)	−0.0088 (13)
C26	0.0240 (16)	0.0212 (14)	0.0385 (17)	0.0126 (12)	0.0051 (13)	−0.0039 (12)
C27	0.0223 (15)	0.0208 (14)	0.0263 (14)	0.0099 (11)	0.0048 (12)	0.0016 (11)
C28	0.0138 (13)	0.0217 (13)	0.0199 (13)	0.0086 (10)	−0.0004 (10)	0.0011 (10)
C29	0.0213 (15)	0.0225 (14)	0.0253 (14)	0.0093 (11)	0.0012 (12)	0.0029 (11)
C30	0.0240 (16)	0.0310 (16)	0.0291 (15)	0.0107 (12)	0.0035 (12)	0.0120 (12)
C31	0.0245 (16)	0.0416 (18)	0.0193 (14)	0.0123 (13)	0.0030 (12)	0.0043 (13)
C32	0.0227 (16)	0.0311 (16)	0.0235 (14)	0.0098 (12)	−0.0008 (12)	−0.0044 (12)
C33	0.0158 (14)	0.0211 (13)	0.0239 (14)	0.0048 (10)	−0.0005 (11)	0.0018 (11)
C34	0.0166 (14)	0.0189 (13)	0.0217 (13)	0.0030 (10)	0.0004 (11)	0.0019 (10)
C35	0.0223 (15)	0.0199 (13)	0.0263 (14)	0.0024 (11)	0.0000 (12)	0.0043 (11)
C36	0.0252 (16)	0.0254 (15)	0.0311 (16)	−0.0005 (12)	−0.0010 (13)	0.0102 (12)
C37	0.0265 (17)	0.0427 (19)	0.0253 (15)	0.0014 (14)	0.0063 (13)	0.0104 (13)
C38	0.0249 (17)	0.0377 (17)	0.0284 (16)	0.0066 (13)	0.0074 (13)	−0.0002 (13)
C39	0.0206 (15)	0.0217 (14)	0.0220 (14)	0.0049 (11)	0.0010 (11)	0.0018 (11)
C40	0.0209 (14)	0.0139 (12)	0.0203 (13)	0.0128 (10)	0.0021 (11)	0.0004 (10)
C41	0.0250 (15)	0.0174 (13)	0.0241 (14)	0.0118 (11)	0.0012 (12)	−0.0004 (11)
C42	0.0288 (17)	0.0235 (14)	0.0270 (15)	0.0123 (12)	0.0103 (13)	0.0013 (12)
C43	0.0383 (18)	0.0220 (14)	0.0209 (14)	0.0157 (13)	0.0024 (13)	0.0002 (11)
C44	0.0293 (17)	0.0169 (13)	0.0212 (14)	0.0084 (11)	−0.0051 (12)	−0.0023 (11)
C45	0.0215 (15)	0.0161 (13)	0.0258 (14)	0.0092 (11)	0.0027 (12)	0.0002 (11)
C46	0.0171 (14)	0.0173 (13)	0.0221 (13)	0.0066 (10)	0.0018 (11)	−0.0002 (10)
C47	0.0205 (15)	0.0182 (13)	0.0288 (15)	0.0055 (11)	0.0031 (12)	0.0026 (11)
C48	0.0245 (16)	0.0181 (13)	0.0286 (15)	0.0068 (11)	0.0003 (12)	−0.0043 (11)
C49	0.0214 (15)	0.0192 (13)	0.0226 (13)	0.0091 (11)	0.0017 (11)	−0.0029 (10)
C50	0.0174 (14)	0.0171 (12)	0.0193 (13)	0.0091 (10)	0.0016 (10)	0.0003 (10)
C51	0.0177 (14)	0.0149 (12)	0.0227 (13)	0.0056 (10)	0.0036 (11)	−0.0008 (10)
C52	0.0247 (16)	0.0199 (13)	0.0216 (13)	0.0082 (11)	0.0054 (11)	0.0001 (11)
C53	0.0284 (16)	0.0193 (13)	0.0235 (14)	0.0069 (11)	0.0011 (12)	0.0047 (11)
C54	0.0276 (16)	0.0137 (12)	0.0293 (15)	0.0098 (11)	0.0011 (12)	0.0022 (11)
C55	0.0220 (15)	0.0194 (13)	0.0214 (13)	0.0083 (11)	0.0014 (11)	−0.0022 (11)
C1S	0.0308 (18)	0.0207 (14)	0.0379 (17)	0.0084 (12)	0.0032 (14)	−0.0041 (13)
Cl1	0.0544 (6)	0.0254 (4)	0.0350 (4)	−0.0024 (4)	0.0083 (4)	0.0012 (3)
Cl2	0.0340 (5)	0.0375 (5)	0.0610 (6)	0.0136 (4)	0.0110 (4)	−0.0071 (4)

### Geometric parameters (Å, °)

**Table:**

Ni1—N2	2.006 (2)	C22—C27	1.404 (4)
Ni1—N8	2.024 (2)	C23—C24	1.384 (4)
Ni1—N7	2.032 (2)	C23—H23	0.9500
Ni1—N6	2.072 (2)	C24—C25	1.390 (4)
Ni1—N4	2.078 (2)	C24—H24	0.9500
B1—N5	1.553 (4)	C25—C26	1.381 (4)
B1—N3	1.560 (4)	C25—H25	0.9500
B1—N1	1.562 (4)	C26—C27	1.385 (4)
B1—H1	1.0000	C26—H26	0.9500
N1—C3	1.362 (3)	C27—H27	0.9500
N1—N2	1.371 (3)	C28—C29	1.402 (4)
N2—C1	1.346 (3)	C28—C33	1.405 (4)
N3—C6	1.360 (3)	C29—C30	1.384 (4)
N3—N4	1.369 (3)	C29—H29	0.9500
N4—C4	1.350 (4)	C30—C31	1.398 (4)
N5—C9	1.374 (3)	C30—H30	0.9500
N5—N6	1.381 (3)	C31—C32	1.389 (4)
N6—C7	1.345 (3)	C31—H31	0.9500
N7—C50	1.335 (3)	C32—C33	1.389 (4)
N7—C46	1.354 (3)	C32—H32	0.9500
N8—C51	1.345 (3)	C33—H33	0.9500
N8—C55	1.362 (4)	C34—C39	1.391 (4)
N9—C50	1.376 (3)	C34—C35	1.402 (4)
N9—C51	1.385 (3)	C35—C36	1.392 (4)
N9—H9	0.8800	C35—H35	0.9500
C1—C2	1.395 (4)	C36—C37	1.376 (5)
C1—C10	1.488 (4)	C36—H36	0.9500
C2—C3	1.383 (4)	C37—C38	1.387 (5)
C2—H2	0.9500	C37—H37	0.9500
C3—C16	1.482 (4)	C38—C39	1.393 (4)
C4—C5	1.392 (4)	C38—H38	0.9500
C4—C22	1.477 (4)	C39—H39	0.9500
C5—C6	1.390 (4)	C40—C41	1.397 (4)
C5—H5	0.9500	C40—C45	1.397 (4)
C6—C28	1.474 (4)	C41—C42	1.393 (4)
C7—C8	1.394 (4)	C41—H41	0.9500
C7—C34	1.476 (4)	C42—C43	1.390 (4)
C8—C9	1.384 (4)	C42—H42	0.9500
C8—H8	0.9500	C43—C44	1.381 (4)
C9—C40	1.473 (4)	C43—H43	0.9500
C10—C15	1.392 (4)	C44—C45	1.395 (4)
C10—C11	1.396 (3)	C44—H44	0.9500
C11—C12	1.390 (4)	C45—H45	0.9500
C11—H11	0.9500	C46—C47	1.370 (4)
C12—C13	1.385 (4)	C46—H46	0.9500
C12—H12	0.9500	C47—C48	1.397 (4)
C13—C14	1.387 (4)	C47—H47	0.9500
C13—H13	0.9500	C48—C49	1.373 (4)
C14—C15	1.394 (4)	C48—H48	0.9500
C14—H14	0.9500	C49—C50	1.407 (3)
C15—H15	0.9500	C49—H49	0.9500
C16—C21	1.388 (4)	C51—C52	1.403 (4)
C16—C17	1.390 (4)	C52—C53	1.378 (4)
C17—C18	1.392 (4)	C52—H52	0.9500
C17—H17	0.9500	C53—C54	1.392 (4)
C18—C19	1.389 (5)	C53—H53	0.9500
C18—H18	0.9500	C54—C55	1.368 (4)
C19—C20	1.391 (5)	C54—H54	0.9500
C19—H19	0.9500	C55—H55	0.9500
C20—C21	1.390 (4)	C1S—Cl1	1.762 (3)
C20—H20	0.9500	C1S—Cl2	1.777 (3)
C21—H21	0.9500	C1S—H1S1	0.9900
C22—C23	1.397 (4)	C1S—H1S2	0.9900
			
N2—Ni1—N8	102.80 (9)	C23—C22—C4	122.1 (2)
N2—Ni1—N7	93.15 (8)	C27—C22—C4	119.5 (3)
N8—Ni1—N7	88.45 (9)	C24—C23—C22	121.1 (3)
N2—Ni1—N6	89.55 (8)	C24—C23—H23	119.5
N8—Ni1—N6	96.98 (9)	C22—C23—H23	119.5
N7—Ni1—N6	173.26 (9)	C23—C24—C25	120.0 (3)
N2—Ni1—N4	97.52 (9)	C23—C24—H24	120.0
N8—Ni1—N4	159.63 (9)	C25—C24—H24	120.0
N7—Ni1—N4	89.09 (9)	C26—C25—C24	119.4 (3)
N6—Ni1—N4	84.44 (9)	C26—C25—H25	120.3
N5—B1—N3	106.6 (2)	C24—C25—H25	120.3
N5—B1—N1	110.5 (2)	C25—C26—C27	121.1 (3)
N3—B1—N1	109.2 (2)	C25—C26—H26	119.4
N5—B1—H1	110.2	C27—C26—H26	119.4
N3—B1—H1	110.2	C26—C27—C22	120.0 (3)
N1—B1—H1	110.2	C26—C27—H27	120.0
C3—N1—N2	109.4 (2)	C22—C27—H27	120.0
C3—N1—B1	132.0 (2)	C29—C28—C33	118.7 (2)
N2—N1—B1	118.5 (2)	C29—C28—C6	118.9 (2)
C1—N2—N1	107.1 (2)	C33—C28—C6	122.4 (2)
C1—N2—Ni1	133.80 (17)	C30—C29—C28	120.8 (3)
N1—N2—Ni1	113.87 (16)	C30—C29—H29	119.6
C6—N3—N4	109.7 (2)	C28—C29—H29	119.6
C6—N3—B1	132.2 (2)	C29—C30—C31	120.0 (3)
N4—N3—B1	117.9 (2)	C29—C30—H30	120.0
C4—N4—N3	107.0 (2)	C31—C30—H30	120.0
C4—N4—Ni1	136.90 (18)	C32—C31—C30	119.8 (3)
N3—N4—Ni1	115.86 (16)	C32—C31—H31	120.1
C9—N5—N6	108.8 (2)	C30—C31—H31	120.1
C9—N5—B1	130.7 (2)	C33—C32—C31	120.4 (3)
N6—N5—B1	119.5 (2)	C33—C32—H32	119.8
C7—N6—N5	107.3 (2)	C31—C32—H32	119.8
C7—N6—Ni1	139.13 (18)	C32—C33—C28	120.3 (3)
N5—N6—Ni1	113.57 (15)	C32—C33—H33	119.8
C50—N7—C46	118.4 (2)	C28—C33—H33	119.8
C50—N7—Ni1	122.66 (17)	C39—C34—C35	118.6 (3)
C46—N7—Ni1	118.26 (16)	C39—C34—C7	121.2 (2)
C51—N8—C55	117.2 (2)	C35—C34—C7	120.1 (3)
C51—N8—Ni1	121.62 (19)	C36—C35—C34	120.5 (3)
C55—N8—Ni1	119.78 (18)	C36—C35—H35	119.8
C50—N9—C51	129.6 (2)	C34—C35—H35	119.8
C50—N9—H9	115.2	C37—C36—C35	120.0 (3)
C51—N9—H9	115.2	C37—C36—H36	120.0
N2—C1—C2	109.6 (2)	C35—C36—H36	120.0
N2—C1—C10	121.9 (2)	C36—C37—C38	120.4 (3)
C2—C1—C10	128.5 (2)	C36—C37—H37	119.8
C3—C2—C1	106.1 (2)	C38—C37—H37	119.8
C3—C2—H2	127.0	C37—C38—C39	119.8 (3)
C1—C2—H2	127.0	C37—C38—H38	120.1
N1—C3—C2	107.8 (2)	C39—C38—H38	120.1
N1—C3—C16	124.3 (2)	C34—C39—C38	120.7 (3)
C2—C3—C16	127.9 (2)	C34—C39—H39	119.7
N4—C4—C5	109.6 (2)	C38—C39—H39	119.7
N4—C4—C22	122.3 (2)	C41—C40—C45	118.9 (2)
C5—C4—C22	128.1 (3)	C41—C40—C9	123.7 (2)
C6—C5—C4	106.1 (2)	C45—C40—C9	117.3 (2)
C6—C5—H5	127.0	C42—C41—C40	120.3 (3)
C4—C5—H5	127.0	C42—C41—H41	119.8
N3—C6—C5	107.6 (2)	C40—C41—H41	119.8
N3—C6—C28	124.7 (2)	C43—C42—C41	120.0 (3)
C5—C6—C28	127.7 (3)	C43—C42—H42	120.0
N6—C7—C8	109.8 (2)	C41—C42—H42	120.0
N6—C7—C34	122.6 (2)	C44—C43—C42	120.4 (3)
C8—C7—C34	127.6 (2)	C44—C43—H43	119.8
C9—C8—C7	106.3 (2)	C42—C43—H43	119.8
C9—C8—H8	126.8	C43—C44—C45	119.7 (3)
C7—C8—H8	126.8	C43—C44—H44	120.1
N5—C9—C8	107.8 (2)	C45—C44—H44	120.1
N5—C9—C40	125.3 (2)	C44—C45—C40	120.7 (3)
C8—C9—C40	126.8 (2)	C44—C45—H45	119.6
C15—C10—C11	119.2 (2)	C40—C45—H45	119.6
C15—C10—C1	120.6 (2)	N7—C46—C47	123.5 (3)
C11—C10—C1	120.1 (2)	N7—C46—H46	118.2
C12—C11—C10	120.1 (3)	C47—C46—H46	118.2
C12—C11—H11	120.0	C46—C47—C48	117.8 (3)
C10—C11—H11	120.0	C46—C47—H47	121.1
C13—C12—C11	120.4 (3)	C48—C47—H47	121.1
C13—C12—H12	119.8	C49—C48—C47	119.6 (3)
C11—C12—H12	119.8	C49—C48—H48	120.2
C12—C13—C14	120.0 (3)	C47—C48—H48	120.2
C12—C13—H13	120.0	C48—C49—C50	119.0 (3)
C14—C13—H13	120.0	C48—C49—H49	120.5
C13—C14—C15	119.7 (3)	C50—C49—H49	120.5
C13—C14—H14	120.1	N7—C50—N9	120.3 (2)
C15—C14—H14	120.1	N7—C50—C49	121.5 (2)
C10—C15—C14	120.6 (3)	N9—C50—C49	118.2 (2)
C10—C15—H15	119.7	N8—C51—N9	120.6 (2)
C14—C15—H15	119.7	N8—C51—C52	122.3 (2)
C21—C16—C17	119.1 (3)	N9—C51—C52	117.1 (2)
C21—C16—C3	121.2 (3)	C53—C52—C51	118.8 (3)
C17—C16—C3	119.5 (3)	C53—C52—H52	120.6
C16—C17—C18	120.7 (3)	C51—C52—H52	120.6
C16—C17—H17	119.7	C52—C53—C54	119.4 (3)
C18—C17—H17	119.7	C52—C53—H53	120.3
C19—C18—C17	119.9 (3)	C54—C53—H53	120.3
C19—C18—H18	120.0	C55—C54—C53	118.4 (3)
C17—C18—H18	120.0	C55—C54—H54	120.8
C18—C19—C20	119.6 (3)	C53—C54—H54	120.8
C18—C19—H19	120.2	N8—C55—C54	123.7 (3)
C20—C19—H19	120.2	N8—C55—H55	118.1
C21—C20—C19	120.1 (3)	C54—C55—H55	118.1
C21—C20—H20	120.0	Cl1—C1S—Cl2	111.10 (16)
C19—C20—H20	120.0	Cl1—C1S—H1S1	109.4
C16—C21—C20	120.6 (3)	Cl2—C1S—H1S1	109.4
C16—C21—H21	119.7	Cl1—C1S—H1S2	109.4
C20—C21—H21	119.7	Cl2—C1S—H1S2	109.4
C23—C22—C27	118.4 (2)	H1S1—C1S—H1S2	108.0

### Hydrogen-bond geometry (Å, °)

**Table:**

*D*—H···*A*	*D*—H	H···*A*	*D*···*A*	*D*—H···*A*
N9—H9···Br1^i^	0.88	2.41	3.265 (2)	165
C52—H52···Br1^i^	0.95	2.92	3.706 (3)	140
C31—H31···Br1^ii^	0.95	2.91	3.858 (3)	172

Symmetry codes: (i) −*x*+1, −*y*+1, −*z*+1; (ii) −*x*+1, −*y*+1, −*z*+2.
